# Expression plasticity of *Phlebotomus papatasi *salivary gland genes in distinct ecotopes through the sand fly season

**DOI:** 10.1186/1472-6785-11-24

**Published:** 2011-10-10

**Authors:** Iliano V Coutinho-Abreu, Rami Mukbel, Hanafi A Hanafi, Emad Y Fawaz, Shabaan S El-Hossary, Mariha Wadsworth, Gwen Stayback, Dilkushi A Pitts, Mahmoud Abo-Shehada, David F Hoel, Shaden Kamhawi, Marcelo Ramalho-Ortigão, Mary Ann McDowell

**Affiliations:** 1 The Eck Institute for Global Health, Department of Biological Sciences, University of Notre Dame, Notre Dame, IN, 46556, USA; 2 Research Sciences Directorate, U.S. Naval Medical Research Unit No. 3 (NAMRU-3), PSC 452, Box 5000, FPO AE 09863-007, USA; 3 Center for Research Computing and Department of Civil and Geological Sciences, University of Notre Dame, Notre Dame, IN, 46556, USA; 4 Faculty of Veterinary Medicine, Jordan University of Science and Technology, Irbid, 22110 Jordan; 5 Laboratory of Malaria and Vector Research, NIAID-NIH, 12735 Twinbrook Parkway, Rockville, MD 20852, USA; 6 Department of Entomology, Kansas State University, 123 W. Waters Hall, Kansas State University, Manhattan KS, USA; 7 Center for Disease Control, 1600 Clifton Road, Atlanta, GA 30329, USA

## Abstract

**Background:**

Sand fly saliva can drive the outcome of *Leishmania *infection in animal models, and salivary components have been postulated as vaccine candidates against leishmaniasis. In the sand fly *Phlebotomus papatasi*, natural sugar-sources modulate the activity of proteins involved in meal digestion, and possibly influence vectorial capacity. However, only a handful of studies have assessed the variability of salivary components in sand flies, focusing on the effects of environmental factors in natural habitats. In order to better understand such interactions, we compared the expression profiles of nine *P. papatasi *salivary gland genes of specimens inhabiting different ecological habitats in Egypt and Jordan and throughout the sand fly season in each habitat.

**Results:**

The majority of investigated genes were up-regulated in specimens from Swaymeh late in the season, when the availability of sugar sources is reduced due to water deprivation. On the other hand, these genes were not up-regulated in specimens collected from Aswan, an irrigated area less susceptible to drought effects.

**Conclusion:**

Expression plasticity of genes involved with vectorial capacity in disease vectors may play an important epidemiological role in the establishment of diseases in natural habitats.

## Background

Many studies have demonstrated an environmental role in gene expression. Differential gene expression can be caused by biotic (e.g., virus infections and menopause in humans) or abiotic factors (e.g., arsenic poisoning and diesel exposure in humans; or temperature variation in worms and plants). Effects of the environment on gene expression are referred to as gene-by-environment-interactions, and the response displayed by organisms to such environmental change, phenotypic plasticity [1]. Although much is known about the ecology of the sand fly *Phlebotomus papatasi *[2-9], how the environment influences gene expression in this insect remains largely unexplored [10].

*Phlebotomus papatasi *is the primary vector of *Leishmania major *in Northern Africa and the Middle East [11,12]. The behavior of this sand fly species is well documented with regards to resting places [12], blood sources [13] and dispersal ability [9]. In addition to blood, sugar also constitutes a key component of the sand fly life cycle, and several plant species are able to attract sand flies. In the Middle East, *Prosopis farcta*, *Capparis spinosa*, *Ricinus communis*, *Solanum nigrum*, and *Rochia indica *are some of the most attractive plants for *P. papatasi *[2]. Analyses of sugar contents in the gut of field caught flies revealed that 15.5% of the flies were fed on some type of sugar, and 22.5% of them presented cellulose shreds within their guts. Starch is also an important nutrient for *P. papatasi*, as demonstrated by the finding that 50% of the field collected flies had ingested this carbohydrate [5]. Starch is likely obtained from the sap of the succulent plant *Atriplex halimus *[5], which is frequently associated with burrows of the *L. major *reservoir host, the fat sand rat *Psammomys obesus *[2].

Previous studies have demonstrated that sugar appears to influence many aspects of sand fly physiology, including longevity [3,14]. Whereas only 2.6% *P. papatasi *females collected in non-irrigated areas in the Jordan Valley are older than 8 days [3], in irrigated regions where sugar-rich sources are available the longevity of flies is much greater, averaging 33 days [5]. Accordingly, estimates of sugar content in three species of plants inhabiting non-irrigated areas are more than 3 fold lower than in the same species from irrigated habitats [7].

The interaction of sugars with various aspects of sand fly physiology and *Leishmania *development is not yet fully understood. Recent studies have contributed to our understanding of some of these complex interactions. Although a sugar-rich diet is associated with a greater number of gonotrophic cycles for *P. papatasi *(i.e., up to 5 cycles), thus, increasing the chance for *Leishmania *transmission [5], feeding on plants, such as *R. communis*, *C. spinosa*, and *S. luteum *can actually decrease the number of *Leishmania *in *P. papatasi *by 45% [3]. In addition, an eight fold decrease in *Leishmania *load in the gut of sand flies was found when they fed on *Malva nicaeensis *compared to *A. halimus *[6,7].

The quality of the sugar meals is also believed to influence sugar-feeding behavior of *P. papatasi *[5]. Such effects appear to be driven by the rate of photosynthesis and the quality of the sugars produced by a given plant species. These data are supported by the observation that *P. papatasi *prefers feeding on branches of the Syrian mesquite *P. farcta *collected from a humid habitat rather than on branches of the same plant collected from a dry and salty soil [5].

The expression of some sand fly genes involved in digestion and nutrient acquisition are also modulated by sugar-meals. Chitinases of the sand flies *P. papatasi *and *L. longipalpis *s.l. are not expressed after a sugar meal [15], whereas two putative trypsin encoding genes, *PperTryp1 and 1*, a chymotrypsin, *PperChym3*, are up-regulated when *P. perniciosus *is sugar-fed [16]. Likewise, *L. longipalpis *s.l. salivary protein content is also increased after a sugar-meal [17].

Saliva components have been identified for several insect species including sand flies [18-22]. Like that of many other blood sucking insects, sand fly saliva was shown to play roles in vasodilation as well as in inhibiting blood clotting and platelet aggregation [22], though the molecules utilized for such tasks may vary between different sand fly genera. For instance, in the New World sand fly *L. longipalpis s.l*. a 6 KDa peptide named maxadilan is responsible for the vasodilatory effect [22]. In *P. papatasi*, this is accomplished by adenosine and 5'AMP [22]. Other functions, however, are performed by molecules conserved between the two genera [22]. Sand flies have evolved a cocktail of salivary components to overcome the complex hemostasis system of their hosts [23]. A diversity of compounds may be required for these bloodsucking animals to disarm the redundant barriers presented by their hosts or for feeding on multiple host species.

Sand fly saliva is essential for the success of *Leishmania *transmission as it is necessary for successful blood feeding. Interestingly, *P. papatasi *saliva exacerbates *L. major *infection in mice [24]; however, pre-exposure to sand fly saliva or non-infected sand fly bites confers protection against lesion development in the same animals [25], suggesting that sand fly saliva could potentially serve as a component in a vaccine against leishmanial disease. Moreover, protection in mice can be achieved by pre-vaccination of animals with a plasmid encoding a 15 kDa protein (*SP15*) present in the sialome of *P. papatasi *[21], though protection is mediated by different antigens in different hosts [21,25-27]. Before sand fly saliva can be fully exploited as a vaccine target, the genetic and expression variability of salivary proteins must first be assessed in the field.

The most highly expressed proteins in the sialome from *P. papatasi *have been identified, and those encompass the products of 12 genes [21]; however, no complete transcriptome data is available. These genes are continuously expressed after adult hatching [28], and their protein product levels peaks at three days post-hatching [29]. Recently, it was suggested that various enzyme activities associated with *P. papatasi *vectorial capacity are differentially modulated in distinct ecological habitats [10]. Here, we analyzed the gene expression plasticity of nine *P. papatasi *salivary gland genes across specimens collected in distinct ecotopes and obtained during different periods during the sand fly season. Our results indicate that the pattern of salivary gland gene expression exhibited is more associated with the distinct environmental conditions presented in natural habitats than with the geographic origins of the specimens. Additionally, the data presented support the notion that expression plasticity of sand fly salivary gland genes exhibited in distinct ecological habitats may have epidemiological consequences and may affect the immunogenicity of a sand fly salivary protein-based vaccine.

## Methods

### Sand flies

*Phlebotomus papatasi *used in this study were either obtained from field collections or from a colony (Israeli strain - PPIS) maintained at the University of Notre Dame. These PPIS specimens are from a colony originally established in the mid 1970's that went through several bottlenecks, the most current of which was in July 2007. Thus, the PPIS colony displays very low levels of genetic polymorphism. For field samples, sand flies were collected at 3 locations: Aswan (GPS coordinates N 24°10, E 32°52), in a village adjacent to the Nile River (Baharif - Southern Egypt); Northern Sinai (GPS coordinates N 30°50', E 34°10'), in a Bedouin village (Om Shikhan - Northeastern Egypt); and Swaymeh (GPS coordinates N 31°48', E 35°35'), near the Dead Sea, in Jordan.

The collection site in Aswan, Baharif village, is located on the east margin of the Nile. This village is typically cultivated with date palms (*Phoenix dactylifera*), mangoes (*Mangifera indica*), wheat (*Triticum aestivum*), corn (*Zea mays*), and clover (*Trifolium *spp.) under artificial irrigation. The human population is approximately 400 and the village is stocked with domestic animals including cattle, dogs, and goats. Daily temperatures typically range from 24°C to 45°C, and it seldom rains in this locality. This site was chosen for our study because of the large number of sand flies present in the area as observed by U.S. Naval Medical Research Unit No. 3 (NAMRU-3) researchers over the previous 15 years, for the absence of *Leishmania*-infected flies [30], and because it is an irrigated area.

In North Sinai, sand flies were collected in Om Shikhan, located approximately 340 km east of Cairo, 80 km inland from the Mediterranean coast, and 30 km west of the Israeli border in North Sinai, Egypt. The area terrain is typical rolling sand desert with sufficient rainfall and humidity to permit cultivation of fruit trees, melon, and millet by the local Bedouin population. This area is unique in having a heightened water table, produced by the nearby (3 km distant) El Ruafa Dam, on Wadi El Arish. Uncultivated areas around the reservoir are variably covered by low desert brush, with *Artemisia, Panicum, Salicornia*, Tamarisk and *Thymelaea *predominating [31]. Climatic conditions produce a mean precipitation of 87 mm per year, with summer mean daily maximum temperature of 33.5°C, and winter mean daily minimum of 6°C. This collection site is an endemic site for *L. major *infections.

Swaymeh, Jordan, is an area of low elevation at approximately 350 m below sea level. The climate is considered Saharan Mediterranean with temperatures ranging from a minimum of 8-12°C in the winter and a maximum of 35-40°C in the summer. Mean rainfall is <50 mm, with all rain fall ocurring from November-April. Area soil is mostly sandy or sandy hammada with granite fragments and saline, with tropical and halophytic vegetation (chenopods such as *Atriplex halimu*s and *Suaeda *spp.) as the natural flora. Swaymeh also is an endemic area for *L. major*.

Whenever possible, sand fly trappings were carried out three times a year, early (June), middle (August) and late (September) for years 2006 and 2007. While in Aswan and Swaymeh we performed 3 trappings (late 06, early and mid 07); for the sites in North Sinai only 2 trappings took place: early and mid 07 in North Sinai. For each of the 3 locations *P. papatasi *represents approximately 95% of the *Phlebotomus *species [30,32]. Sand flies were trapped using CDC-style light traps between 18:00 and 06:00. Traps were either baited with CO_2 _(dry ice) (for trappings done in Aswan and North Sinai), or non-baited (Swaymeh). Sand flies were transferred from collection bags and maintained alive until dissected. Flies were euthanized in water and detergent just prior to dissection. *P. papatasi *were identified by microscopic examination of female spermateca according to Lane [33]; their heads along with the salivary glands were pulled off the bodies, transferred to 50 μl of RNAlater™ solution (Ambion, Austin, TX, USA), homogenized, and stored at -20°C.

### Environmental and landscape data

The Normalized Difference Vegetation Index (NDVI) was used as a measure of healthy vegetation cover [34]. NDVI is one of the simplest and most commonly used indices for ecological assessment [35-38]. This index has shown reasonable correlation with vegetation abundance [36] and other important ecological parameters such as leaf area index (LAI) values [39,40]. A 10-day composite map of NDVI data for mid September 2006 was downloaded from http://www.fao.org/giews/english/windisp/data.htm and clipped using the boundaries of Egypt and Jordan.

Digital elevation data also were downloaded for Egypt http://earlywarning.usgs.gov/fews/africa/index.php and Jordan http://www.eeaa.gov.eg/English/main/about.asp. The digital elevation maps also were clipped using the boundaries of Egypt and Jordan.

### RNA extraction and cDNA synthesis

The RNA was extracted from the dissected tissues (head and salivary glands) of all the *P. papatasi *individually using the RNAeasy Mini Kit (Qiagen, Valencia, CA, USA) according to instructions and stored at -80°C.

cDNAs were synthesized using Invitrogen reagents (Invitrogen, Carlsbad, CA, USA), following the manufacturer's instructions. In brief, 12 μl RNA from each sample were added with 2.5 μM Oligo (dT)_20 _primer and 0.5 μM dNTPs (10 mM), incubated at 65°C for 5 minutes (min) and kept in ice for at least 1 min; 4 μl 5 × SuperScript™ III Reverse Transcriptase First-Strand Buffer, 5 mM DTT (0.1 M), 20 Units of RNase OUT, and 200 Units of SuperScript™ III Reverse Transcriptase (200 u/μl) were added to the reaction. The mixture was incubated for one hour at 50°C and stored at -20°C.

### Real-time polymerase chain reactions (RT-PCR)

RT-PCR reactions were set up with 10 μl SYBR Green reagent (Applied Biosystems, Foster City, CA, USA), 0.6 μl each forward and reverse primer (0.3 μM final concentration), 0.5 μl each cDNA sample, and 8.3 μl Ultra Pure DNase/RNase-Free Water (Invitrogen). Reactions were analyzed in 96-well plate format using a 7900 HT Fast Real Time PCR System (Applied Biosystems) under the following conditions: initial incubation at 50°C for 2 min and 95°C for 10 min; followed by 40 cycles of 95°C for 15 sec, 55°C for 1 min; ending with a dissociation step of 95°C for 15 sec, 55°C for 15 sec, and 95°C for 15 sec.

The 9 *P. papatasi *salivary gland genes assayed for expression in this study are *SP12*, *SP14, SP28, SP29, SP30, SP32, SP36, SP42*, and *SP44*. The primers used in the RT-PCR reactions were published elsewhere [28]. α-tubulin was used as a housekeeping load control [41]. A total of 20 *P. papatasi *specimens from each field catch were used for individual RT-PCR reactions, and the expression profile for all 9 cDNAs was assessed using the same 20 field-caught samples. Each reaction was repeated four times for each gene (twice in two different plates) for a total of 80 reactions per sand fly trapping. Since the expression analyses of nine genes in eight different sand fly trappings were performed, a total of 5760 RT-PCR reactions were performed during the completion of this study.

Differential expression results for each salivary protein gene were displayed as fold changes over a control, using the 2^-ΔΔCT ^method [42]. The fold changes were calculated by the expression 2^-ΔΔCT^, where ΔΔC_T _= ΔC_T_(sample) - ΔC_T_(calibrator), ΔC_T _= ΔC_T_(sample) -ΔC_T_(alpha tubulin gene), C_T _= cycle at which a statistically significant increase in the emission intensity over the background. The calibrator was represented by the average expression (mean ΔC_T_) of the seven non-fed samples (PPIS) dissected 24 hours after emerging [28]. Fold changes were calculated for each sample.

### Statistical analysis

Statistical analyses were carried out using the software GraphPad Prism v. 5.01 (GraphPad Software, Inc). The statistical tests used were the non-parametric Kruskal-Wallis tests, for comparisons among more than two data sets, and/or Mann-Whitney tests, for pairwise comparisons between data sets when the results for Kruskal-Wallis test were statistically significant, or for comparisons when only two data sets were present. The level of significance was adjusted for multiple comparisons using Bonferroni's correction. Differences were considered statistically significant at α = 0.0006. The values of fold change in Tables 1 and 2 are based on the ratio of the expression medians between the time point up-regulated (+) over the time point down-regulated (-).

**Table 1:** *P. papatasi *salivary gland gene expression differences throughout the season

Genes	Regulation of expression			p-values	Fold change
Aswan	Early	x	Middle		

SP30	-		+	p < 0.0001	9.35

	Middle	x	Late		

SP30	+		-	p < 0.0001	5.54

**Genes**	**Regulation of expression**			**p-values**	**Fold change**

Swaymeh	Early	x	Middle		

SP36	-		+	p = 0.0005	3.33

	Early	x	Late		

SP12	-		+	p < 0.0001	2.86

SP29	-		+	p < 0.0001	3.87

SP36	-		+	p < 0.0001	5.51

SP42	-		+	p < 0.0001	4.78

SP44	-		+	p = 0.0004	5.48

	Middle	x	Late		

SP29	-		+	p = 0.0005	2.45

SP42	-		+	p < 0.0001	4.58

SP44	-		+	p < 0.0001	6.33

Only statistically significant (p < 0.0006) differences are shown. Values of fold change are derived from the comparison between expression medians. Regulation of expression levels up (+) or down (-) are indicated.

**Table 2:** Geographic comparisons of *P. papatasi *salivary gland gene expression

Genes	Regulation of expression			p-values	Fold change
Early	Aswan	x	North Sinai		

SP12	-		+	p = 0.0005	2.64

**Genes**	**Regulation of expression**			**p-values**	**Fold change**

Middle	Aswan	x	Swaymeh		

SP28	+		-	p = 0.0001	15.40

SP30	+		-	p = 0.0002	2.65

SP32	-		+	p < 0.0001	24.90

	Aswan	x	North Sinai		

SP30	+		-	p < 0.0001	3.90

	Swaymeh	x	North Sinai		

SP32	+		-	p < 0.0001	8.46

**Genes**	**Regulation of expression**			**p-values**	**Fold change**

Late	Aswan	x	Swaymeh		

SP29	-		+	p < 0.0001	5.33

SP32	-		+	p = 0.0005	7.59

SP36	-		+	p < 0.0001	6.31

SP42	-		+	p < 0.0001	6.73

Only statistically significant (p < 0.0006) differences are shown. Values of fold change are derived from the comparison between expression medians. Regulation of expression levels up (+) or down (-) are indicated.

## Results

**Figure 1:**
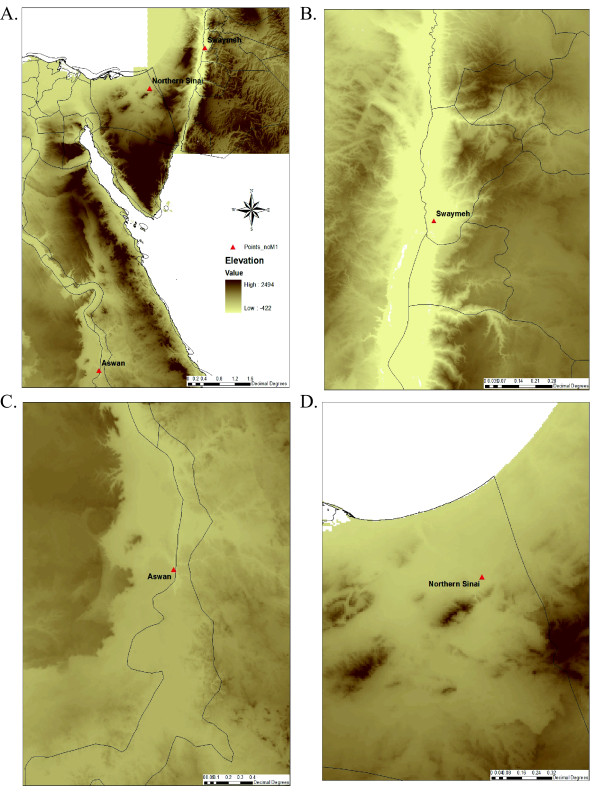
**Elevation maps of study sites**. Elevation maps for entire study region (A), Swaymeh region (B), Aswan region (C), and Northern Sinai region (D).

*P. papatasi *sand flies were collected from three distinct geographic locations. Samples were collected in Aswan, Southern Egypt, as well as in the Northern Sinai Peninsula, Northeastern Egypt. *P. papatasi *were also collected in Swaymeh, Jordan. The elevation of the Egyptial collection sites were 117 m, 141 m above sea level for Aswan and North Sinai, respectively (Figure 1). Swaymeh is located in the Jordan Valley, 345 m below sea level (Figure 1). Swaymeh and North Sinai are ecologically similar habitats, both of which are wetter early in the sand fly season and become drier as the season progresses. Aswan, on the other hand, is an irrigated area adjacent to the Nile River and less influenced by drought effects typical at the end of the sand fly activity season. For each location involved in this study, sand fly trappings were carried out during different periods of the *P. papatasi *activity season [31]. The sand fly season was defined as the period of the year when this sand fly is not overwintering.

**Figure 2:**
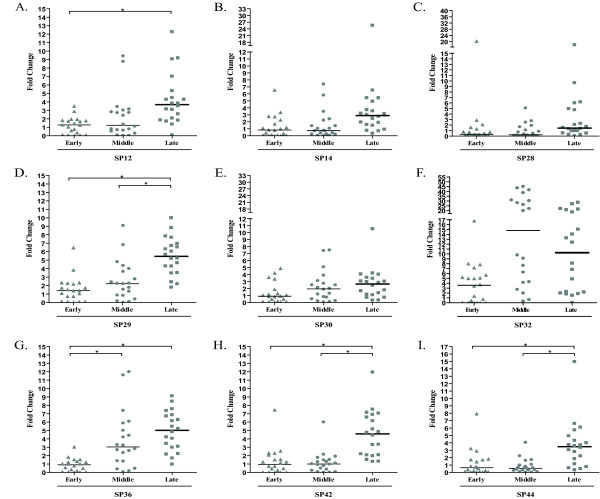
**Salivary Gland Gene Expression from *P. papatasi *collected at Swaymeh**. Expression profiles were assessed as fold changes (Y axis) over the control non-sugar fed, and colony-maintained *P. papatasi *using the 2^-ΔΔCT ^method. Expression profiles of *P. papatasi *collected in June (early in the season) and August (middle) 2007, and September (late) 2006 are displayed for *SP12 *(A), *SP14 *(B), *SP28 *(C), *SP29 *(D), *SP30 *(E), *SP32 *(F), *SP36 *(G), *SP42 *(H), and *SP44 *(I). Horizontal bars represent the expression mean values between the samples and each sample represents an individual fly. Asterisk (*) indicates statistically significant differences (p < 0.0006) between every two groups analyzed. Triangle, circle and square represent expression levels of sand flies collected early, in the middle and late in the season, respectively.

We analyzed the expression of nine out of the 12 most expressed *P. papatasi *salivary gland genes in 20 individual *P. papatasi *collected from each location at each period of the season (early, mid, or late). Two types of analyses were carried out. For the seasonal analyses, gene expression levels were compared between specimens collected at different time points within each habitat (Figure 2; Additional Files 1, 2, 3, 4, 5, 6, 7, 8, and 9, Figures S1-9, A-C, respectively). In the geographic analyses, we compared gene expression levels in specimens collected in different geographic locations, but during the same time period (Additional Files 1, 2, 3, 4, 5, 6, 7, 8, and 9, Figures S1-9, D-E, respectively).

### Seasonal and geographic analyses

Quantitative data of seasonal and geographic analyses are summarized in Tables 1 and 2. For sand flies from Aswan, only the *SP30 *gene is differentially regulated throughout the sand fly season (Table 1; Additional File 5, Figure S5). In sand flies from Swaymeh, five out of the nine genes analyzed (*SP12*, *SP29*, *SP36*, *SP42*, and *SP44*) were differentially regulated through the season (Table 1; Figure 2; Additional Files 1, 4, 7, 8, 9 Figures S1, S4, S7, S8, and S9, respectively). For all 5 genes, we detected an up-regulation of expression towards the driest period of the season (i.e., late in the season; Table 1; Figure 2). For sand flies from North Sinai, none of the nine genes were regulated during the season. However, we were only able to collect at this location in June and August 2007 (Table 1).

Early in the season, only one gene (*SP12*) displayed significantly different levels of expression between the populations of Aswan and North Sinai (Table 2; Additional File 1, Figure S1), being expressed at higher levels in flies from the latter site. Between the populations from Swaymeh and North Sinai, or Swaymeh and Aswan, all the genes analyzed exhibited similar levels of expression (Table 2; Additional Files 1, 2, 3, 4, 5, 6, 7, 8, and 9, Figures S1-9, respectively). At mid season, three genes were differentially expressed between the populations of Aswan and Swaymeh. Two were up-regulated in flies from Aswan (*SP28 *and *SP30*) and one (*SP32*) in individuals from Swaymeh (Table 2; Additional Files 3, 5, 6, Figures S3, S5, and S6, respectively). Between the populations of Aswan and North Sinai, only one gene (*SP30*) was up-regulated in flies from Aswan. Likewise, between Swaymeh and North Sinai one gene was differentially expressed: *SP32 *was up-regulated in Swaymeh (Table 2; Additional File 6, Figure S6). Late in the season, analyses of gene expression revealed four genes (*SP29*, *SP32*, *SP36*, and *SP42*) were up-regulated in Swaymeh in comparison with Aswan (Table 2; Additional Files 4, 6, 7 and 8, Figures S4, S6-8, respectively). As indicated previously, no data was obtained from sand flies in North Sinai late in 2006 (Table 2).

**Figure 3:**
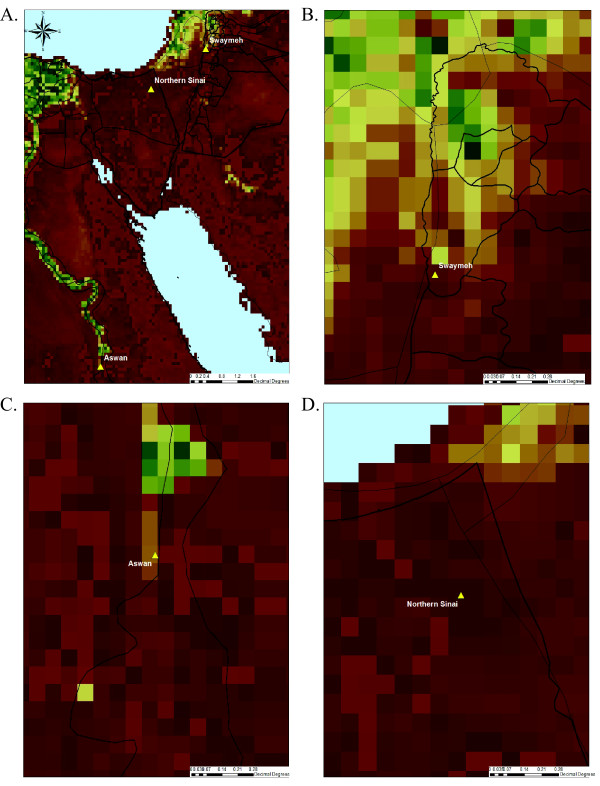
**Vegetation maps of study sites**. Ten day composite maps of NDVI for mid September 2006 for entire study region (A), Swaymeh region (B), Aswan region (C), and Northern Sinai region (D).

As plants are assumed to be the primary source of sugar meals for *P. papatasi *in arid regions [4] and that some sand fly genes are modulated by sugar feeding [15,43], we utilized NVDI to compare the amount of photosynthetic activity between our collection sites during the period (September 2006) when the majority of differences were detected (Figure 3). Although all of the sites would be considered arid, the N. Sinai and Swaymeh sites exhibited NDVI values of 0.083 and 0.098 respectively, values slightly lower than the 0.149 NDVI units calculated for the Aswan site.

## Discussion

The saliva of hematophagous insects plays an essential role in blood feeding. Salivary molecules secreted by blood-sucking arthropods into a vertebrate host overcome the hemostatic system of vertebrates, maintaining blood flow at the site of the bite as well as inhibiting the blood coagulation cascade [22]. In the case of sand flies, saliva also plays a role in the establishment of *Leishmania *infection [24], and salivary proteins are potential vaccine candidates [20,21,25-27,44].

Comparative analyses of salivary protein polymorphisms of sand fly vectors suggest that the development of a vaccine derived from a protein of one species may not protect against *Leishmania *transmitted by a different species [18,45,46] or members of a species complex [47]. However, as protein diversity does not necessarily lead to antigenic polymorphism, protection may be achieved after immunization with salivary proteins of sand flies of the same genera or same species, due to the high degree of similarity exhibited between the salivary proteins of these species [18]. Studies of intra-specific genetic variability further support this hypothesis; low levels of genetic differentiation were displayed between the salivary protein encoding genes of two *P. duboscqi *populations. The similarities between protein sequences ranged from 84-100% while the similarities between their predicted MHC class II binding regions were between 75% and 100% [19]. Furthermore, Elnaiem *et al*. [48] suggested that *P. papatasi *SP15 may be used in a vaccination strategy as their data pointed to a high degree of similarity between different populations, and that the gene was under no selective pressure. Conversely, genetic polymorphisms of maxadilan are antigenically distinct [47]. Intra-species DNA vaccination studies with a specific gene, indicate that cross-species protection is possible [49]. However, protection may be limited to vaccination with specific proteins as only partial species cross reactivity was detected in mice exposed to *P. sergenti *bites [50] and no protection resulted with cross genera challenge in *L. longipalpis s.l*. exposed mice [45].

In addition to genetic variability, expression level differences could influence vaccine efficacy. It recently has been suggested that salivary component expression level differences between laboratory-reared and field-caught sand flies may interfere with the sand fly saliva-mediated protection against *Leishmania *development in mice [51,52]. Our study is the first to investigate differences in the expression profiles of geographically distinct field-collected *P. papatasi *populations and the potential effect of different environments on such profiles. Nine salivary transcripts were analyzed and compared between three different *P. papatasi *populations from the Middle East through three time points during the sand fly season: early (June), mid (August) and late (September). Our study involved the assessment by real time quantitative PCR of 160 individuals and was based on comparisons made to colonized, water-fed only flies. Significant differences in expression levels were found between distinct ecotopes and seasonal periods.

Expression differences of a sand fly salivary gland gene were noted in *L. longipalpis s.l*. [53]. Such differences were used to explain differing erythema sizes caused by bites from *L. longipalpis s.l*. collected from different locations [54]. However, unlike *L. longipalpis s.l*., *P. papatasi *is not a species complex. Nevertheless, several studies have shown physiological, behavioral, and genetic differences between *P. papatasi *populations from different geographic localities [55-59]. Studying colonized sand flies, Wu and Tesh [59] demonstrated that the rate of infection of the *P. papatasi *Israeli strain was higher than sand flies from India or Egypt for infection with *L. major*. Likewise, comparing *P. papatasi *colonies originating from three different populations in Egypt, Hanafi *et al*. [57] indicated that flies from Sinai were more susceptible to *L. major *infection than flies from Aswan or the Nile Delta. Additionally, the feeding rate on mice by *P. papatasi *from Aswan was lower than the rate obtained for the other two populations [58]. In addition to the physiological and behavioral differences, genetic analysis of *P. papatasi *from several colonized and natural populations based on polymorphisms on the *Cytochrome b *(*cyt b*) haplotypes demonstrated moderate genetic differentiation between populations from Egypt and the Middle East [56]. Contrasting these previous studies, analyses of polymorphisms on *P. papatasi Internal Transcribed Spacer 2 *(*ITS2*) and *NAD dehydrogenase subunit 4 *gene (*ND4*) indicated absence of genetic structuring across the *P. papatasi *geographical range [55]. Despite the contrasting information about the genetic structuring of *P. papatasi *populations, gene expression can present a stronger correlation with ecological habitat than with genetic distance [60]. Therefore, *P. papatasi *salivary gland gene expression from field-collected sand flies needs to be thoroughly assessed so that differences observed in flies collected in different ecological habitats can be correlated with the geographic origin of the populations, with seasonal factors present in each habitat, or with both.

To assess whether differences in *P. papatasi *salivary gland gene expression are driven by factors associated with the geographic origin of the populations or by environmental factors (or perhaps both) we performed two types of gene expression comparisons: geographic analyses between specimens collected in different ecological habitats during the same period of the season, and seasonal analyses between specimens collected in the same habitat at different periods of the season. From our analyses, three types of results may be expected: (1) a population displaying higher or lower levels of expression of a given gene for the three geographic comparisons made, but without seasonal differences, indicating that only factors associated with the geographic origin of the populations were responsible for the differences observed in regards to the expression of that specific gene; (2) a population exhibiting higher (or lower) expression medians of a specific gene in the three geographic comparisons as well as in the seasonal analysis, suggesting that both geographic origin-related and seasonal factors drove the expression of that gene; and (3) a population displaying only significant seasonal differences in the expression of a gene, indicating that seasonal environmental factors played a major role in controlling the expression of that gene.

Our geographic analyses indicate that salivary gland genes display expression variability between *P. papatasi *populations from different ecological habitats. Although most of the salivary gland genes from *P. papatasi *collected early in the season exhibited similar levels of expression (Table 2), and only three genes were differentially expressed in the middle of the season (*SP28 *and *SP30*, highly expressed in Aswan; *SP32*, in Swaymeh, Table 2; Figures S3, S5, and S6), late in the season four out of the nine salivary gland genes analyzed displayed greater levels of expression in flies from Swaymeh than from Aswan (Table 2). As none of these populations displayed predominantly higher or lower levels of expression throughout the whole season for all of the genes analyzed, our data suggest that the expression differences of salivary gland genes between *P. papatasi *populations are more influenced by environmental changes during the season in the three localities than by factors associated with the geographic origin the populations studied. The expression levels of four *P. papatasi *salivary gland genes in flies from a dryer habitat (Swaymeh) are higher than in flies from an irrigated area (Aswan) late in the season, when drought may affect sugar content of plants [5].

Phenology studies demonstrated that *P. papatasi *populations from different habitats can exhibit differences in the percentages of gravid or engorged females, as observed in the Jordan Valley [14,32]. However, our own results using laboratory-reared flies indicate that for the genes studied, gravid and engorged females do not exhibit higher levels of expression than sugar fed flies [28].

The seasonal analyses results exhibited here are also similar to *P. papatasi *glycosidase activity patterns presented elsewhere [10]. Five out of nine *P. papatasi *salivary gland genes (*SP12*, *SP29*, *SP36*, *SP42*, and *SP44*) are also differentially regulated during the season (Table 1; Figure 2; Additional Files 1, 4, 7, 8, and 9, Figures S1, S4, and S7-9, respectively). Expression of these *P. papatasi *salivary gland genes was gradually up-regulated reaching the highest levels of expression late in the season in Swaymeh, when the environment is dryer and the sugar sources are scarce (Table 1; Figure 2). In contrast, in a well irrigated area such as Aswan, where drought has little influence on the availability of sugar sources, no late season effect was detected (Table 1). The effect of the low elevation at the Swaymeh site also cannot be ruled out as a factor, as elevation can certainly influence the amount and diversity of vegetation [61]. Furthermore, changes in vegetation also can influence the diversity and abundance of vertebrate hosts available for blood feeding [62,63] and it is possible that the type of blood meal source available could modulate saliva expression patterns. Late season up-regulation effects could not be determined for North Sinai, as sand fly trapping was not possible due to security concerns. Thus, validation of this effect still needs to be demonstrated for sand flies from North Sinai.

Our data suggest that environmental factors play a major role in the expression profiles of *P. papatasi *salivary gland genes. Sap of plants from dry habitats and irrigated areas varies in sugar concentration [7], suggesting that availability of sugar sources is possibly one of the principal factors responsible for the differential expression of salivary gland genes exhibited throughout the season.

Intra-specific comparison of *P. papatasi *salivary gland gene expression between field collected specimens (Additional File 10, Table S1) and colonized flies [28] points to greater variability in species collected in natural habitats. This result may be explained by the greater variety of food sources present in the field that might modulate gene expression in different manners and/or by inbreeding of colonized sand flies.

Schlein and Jacobson [6] demonstrated that *P. papatasi *vectorial capacity in Middle Eastern deserts is linked to hunger tolerance, which is under natural selection. Thus, sand flies from a dry habitat can exhibit greater vectorial capacities than those from an irrigated area [6]. Salivary gland proteins also play a role in *P. papatasi *vectorial capacity as these proteins participate in the establishment of *Leishmania *infection in the vertebrate host [21,24,25]. Accordingly, late in the sand fly activity season, when the expression of some *P. papatasi *salivary gland genes are at their highest level, the number of human cutaneous leishmaniasis (CL) cases is also higher at the Swaymeh site [32] as compared to the beginning of the season. However, in Aswan, where no current cases of CL have been reported, none of the *P. papatasi *salivary gland genes analyzed were up-regulated late in the season. As the incubation period for CL caused by *L. major *ranges between two to eight weeks [64,65], these data further support the notion that the differential gene expression of salivary gland genes exhibited by *P. papatasi *specimens through the season in natural habitats may contribute to the increase in CL cases.

## Conclusions

The use of saliva or salivary components in a multi-component vaccination strategy is a viable option [21,24,25]. However, the geographic and seasonal variation in salivary gland gene expression discovered in this study must be considered in salivary protein-based vaccine development, as antigen dosage is an important component in the modulation of immune responses [66-69] and immunogenicity and specificity of vaccines [66,70,71]. Furthermore, the effect of sand fly saliva induced protection against *Leishmania *may be effected by the amount of exposure the host receives [72].

The genetic plasticity of genes involved with *P. papatasi *vectorial capacity to transmit *L. major*, a parasite responsible for cases of cutaneous leishmaniasis, is evident in field caught specimens, as demonstrated in this work. Moreover, more than half of the salivary gland genes are up-regulated at the end of the sand fly season, when availability of sugar is scarce and disease transmission is increased. Therefore, gene-by-environment interactions can also be an important factor in transmission of pathogens in natural habitats.

## Authors' contributions

IVCA participated in the sand fly collection, performed the real time PCR reactions, analyzed the data, and drafted the manuscript. MAM conceived and coordinated the project and participated in project design, sand fly collections, dissection of salivary glands, and manuscript drafting. MRO participated in sand fly collections, dissection of salivary glands, drafting of the manuscript, and designing of the project. DFH and MAS oversaw operations and sand fly collections in Egypt and Jordan, respectively, including acquiring appropriate permissions and permits when necessary. HAH coordinated all sand fly collections in Egypt and RM and SK coordinated collections in Jordan. EYF and SSE-H participated in all sand fly collections in Egypt and advised on collecting sites. MW and GS maintained the sand fly colony. DAP acquired and formatted the environmental and landscape data. All authors have read and approved the final manuscript.

## Additional Information

The views expressed in this article are those of the author and do not necessarily reflect the official policy or position of the Department of the Navy, Department of Defense, nor the U.S. Government.

The study protocol was approved by the U.S. Naval Medical Research Unit No. 3 Institutional Review Board in compliance with all applicable Federal regulations governing the protection of human subjects. IRB # 193, DoD # NAMRU3.2006.0011.

One of the co-authors is a military service member; some of the other co-authors are employees of the U.S. Government. This work was prepared as part of our official duties. Title 17 U.S.C. §105 provides that 'Copyright protection under this title is not available for any work of the United States Government'. Title 17 U.S.C. §101 defines a U.S. Government work as a work prepared by a military service member or employee of the U.S. Government as part of that person's official duties.

## Supplementary Material

Additional file 1 **Figure S1 - *SP12 *expression**. *SP12 *expression profiles were assessed as fold changes (Y axis) over the control non-sugar fed, and colony-maintained *P. papatasi *using the 2^-ΔΔCT ^method. Seasonal analyses are displayed in (A), (B) and (C), representing the populations of Aswan, Swaymeh and North Sinai, respectively. *P. papatasi *were collected at different periods during the sand fly activity season. Graphs (D), (E) and (F) display the geographic comparisons between expression profiles of *P. papatasi *collected in June (early in the season) and August (middle) 2007, and September (late) 2006, respectively. Horizontal bars represent the expression mean values between the samples and each sample represents an individual fly. Asterisk (*) indicates statistically significant differences (p < 0.0006) between every two groups analyzed. Aswan (black), Swaymeh (dark gray) and North Sinai (light gray) color schemes are shown. Triangle, circle and square represent expression levels of sand flies collected early, in the middle and late in the season, respectively. Click here for file

Additional file 2 **Figure S2 - *SP14 *expression**. *SP14 *expression profiles were assessed as in Figure S1. Seasonal analyses are displayed in (A), (B) and (C), representing the populations of Aswan, Swaymeh and North Sinai, respectively. *P. papatasi *were collected at different periods during the sand fly activity season. Graphs (D), (E) and (F) display the geographic comparisons between expression profiles of *P. papatasi *collected in June (early in the season) and August (middle) 2007, and September (late) 2006, respectively. Horizontal bars represent the expression mean values between the samples, and each sample represents an individual fly. Asterisk (*) indicates statistically significant differences (p < 0.0006) between every two groups analyzed. Aswan (black), Swaymeh (dark gray) and North Sinai (light gray) color schemes are shown. Triangle, circle and square represent expression levels of sand flies collected early, in the middle and late in the season, respectively. Click here for file

Additional file 3 **Figure S3 - *SP28 *expression**. *SP28 *expression profiles were assessed as in Figure S1. Seasonal analyses are displayed in (A), (B) and (C), representing the populations of Aswan, Swaymeh and North Sinai, respectively. *P. papatasi *were collected at different periods during the sand fly activity season. Graphs (D), (E) and (F) display the geographic comparisons between expression profiles of *P. papatasi *collected in June (early in the season) and August (middle) 2007, and September (late) 2006, respectively. Horizontal bars represent the expression mean values between the samples and each sample represents an individual fly. Asterisk (*) indicates statistically significant differences (p < 0.0006) between every two groups analyzed. Aswan (black), Swaymeh (dark gray) and North Sinai (light gray) color schemes are shown. Triangle, circle and square represent expression levels of sand flies collected early, in the middle and late in the season, respectively. Click here for file

Additional file 4 **Figure S4 - *SP29 *expression**. *SP29 *expression profiles were assessed as in Figure S1. Seasonal analyses are displayed in (A), (B) and (C), representing the populations of Aswan, Swaymeh and North Sinai, respectively. *P. papatasi *were collected at different periods during the sand fly activity season. Graphs (D), (E) and (F) display the geographic comparisons between expression profiles of *P. papatasi *collected in June (early in the season) and August (middle) 2007, and September (late) 2006, respectively. Horizontal bars represent the expression mean values between the samples and each sample represents an individual fly. Asterisk (*) indicates statistically significant differences (p < 0.0006) between every two groups analyzed. Aswan (black), Swaymeh (dark gray) and North Sinai (light gray) color schemes are shown. Triangle, circle and square represent expression levels of sand flies collected early, in the middle and late in the season, respectively. Click here for file

Additional file 5 **Figure S5 - *SP30 *expression**. *SP30 *expression profiles were assessed as in Figure S1. Seasonal analyses are displayed in (A), (B) and (C), representing the populations of Aswan, Swaymeh and North Sinai, respectively. *P. papatasi *were collected at different periods during the sand fly activity season. Graphs (D), (E) and (F) display the geographic comparisons between expression profiles of *P. papatasi *collected in June (early in the season) and August (middle) 2007, and September (late) 2006, respectively. Horizontal bars represent the expression mean values between the samples and each sample represents an individual fly. Asterisk (*) indicates statistically significant differences (p < 0.0006) between every two groups analyzed. Aswan (black), Swaymeh (dark gray) and North Sinai (light gray) color schemes are shown. Triangle, circle and square represent expression levels of sand flies collected early, in the middle and late in the season, respectively. Click here for file

Additional file 6 **Figure S6 - *SP32 *expression**. *SP32 *expression profiles were assessed as in Figure S1. Seasonal analyses are displayed in (A), (B) and (C), representing the populations of Aswan, Swaymeh and North Sinai, respectively. *P. papatasi *were collected at different periods during the sand fly activity season. Graphs (D), (E) and (F) display the geographic comparisons between expression profiles of *P. papatasi *collected in June (early in the season) and August (middle) 2007, and September (late) 2006, respectively. Horizontal bars represent the expression mean values between the samples and each sample represents an individual fly. Asterisk (*) indicates statistically significant differences (p < 0.0006) between every two groups analyzed. Aswan (black), Swaymeh (dark gray) and North Sinai (light gray) color schemes are shown. Triangle, circle and square represent expression levels of sand flies collected early, in the middle and late in the season, respectively. Click here for file

Additional file 7 **Figure S7 - *SP36 *expression**. *SP36 *expression profiles were assessed as in Figure S1. Seasonal analyses are displayed in (A), (B) and (C), representing the populations of Aswan, Swaymeh and North Sinai, respectively. *P. papatasi *were collected at different periods during the sand fly activity season. Graphs (D), (E) and (F) display the geographic comparisons between expression profiles of *P. papatasi *collected in June (early in the season) and August (middle) 2007, and September (late) 2006, respectively. Horizontal bars represent the expression median values between the samples and each sample represents an individual fly. Asterisk (*) indicates statistically significant differences (p < 0.0006) between every two groups analyzed. Aswan (black), Swaymeh (dark gray) and North Sinai (light gray) color schemes are shown. Triangle, circle and square represent expression levels of sand flies collected early, in the middle and late in the season, respectively. Click here for file

Additional file 8 **Figure S8 - *SP42 *expression**. *SP42 *expression profiles were assessed as in Figure S1. Seasonal analyses are displayed in (A), (B) and (C), representing the populations of Aswan, Swaymeh and North Sinai, respectively. *P. papatasi *were collected at different periods during the sand fly activity season. Graphs (D), (E) and (F) display the geographic comparisons between expression profiles of *P. papatasi *collected in June (early in the season) and August (middle) 2007, and September (late) 2006, respectively. Horizontal bars represent the expression mean values between the samples and each sample represents an individual fly. Asterisk (*) indicates statistically significant differences (p < 0.0006) between every two groups analyzed. Aswan (black), Swaymeh (dark gray) and North Sinai (light gray) color schemes are shown. Triangle, circle and square represent expression levels of sand flies collected early, in the middle and late in the season, respectively. Click here for file

Additional file 9 **Figure S9 - *SP44 *expression**. *SP44 *expression profiles were assessed as in Figure S1. Seasonal analyses are displayed in (A), (B) and (C), representing the populations of Aswan, Swaymeh and North Sinai, respectively. *P. papatasi *were collected at different periods during the sand fly activity season. Graphs (D), (E) and (F) display the geographic comparisons between expression profiles of *P. papatasi *collected in June (early in the season) and August (middle) 2007, and September (late) 2006, respectively. Horizontal bars represent the expression mean values between the samples and each sample represents an individual fly. Asterisk (*) indicates statistically significant differences (p < 0.0006) between every two groups analyzed. Aswan (black), Swaymeh (dark gray) and North Sinai (light gray) color schemes are shown. Triangle, circle and square represent expression levels of sand flies collected early, in the middle and late in the season, respectively. Click here for file

Additional file 10 **Table S1 - *P. papatasi *salivary gland gene expression**. Median gene expression levels and ranges for all 9 salivary gland genes from each location and collection date are presented. Click here for file
