# Supplementary material for: Expression plasticity of Phlebotomus papatasi salivary gland genes in distinct ecotopes through the sand fly season
Source: BMC Ecol. 2011 Oct 10;11:24. doi: 10.1186/1472-6785-11-24 (PMC3209445; doi:10.1186/1472-6785-11-24)
Supplement: Additional file 10 — Table S1 - P. papatasi salivary gland gene expression. Median gene expression levels and ranges for all 9 salivary gland genes from each location and collection date are presented. [file 1472-6785-11-24-S10.PDF]

Table S1. *P. papatasi* salivary gland gene expression.

| | Aswan | | |
|---|---|---|---|
| | Late | Early | Middle |
| SP12 | 1.59 (0.51-5.53) | 0.85 (0.19-3.09) | 2.02 (0.57-5.03) |
| SP14 | 1.25 (0.24-8.27) | 1.00 (0.18-2.44) | 1.53 (0.36-7.26) |
| SP28 | 1.25 (0.24-29.59) | 0.84 (0.21-3.06) | 3.80 (0.04-13.63) |
| SP29 | 1.02 (0.11-4.65) | 1.35 (0.19-2.35) | 1.53 (0.13-7.16) |
| SP30 | 0.93 (0.10-5.38) | 0.55 (0.05-1.47) | 5.15 (1.17-23.38) |
| SP32 | 1.35 (0.21-8.79) | 1.28 (0.32-8.16) | 0.59 (0.13-3.48) |
| SP36 | 0.79 (0.04-4.34) | 1.05 (0.16-2.39) | 1.86 (0.10-6.46) |
| SP42 | 0.68 (0.16-3.75) | 1.02 (0.25-3.14) | 1.61 (0.21-9.12) |
| SP44 | 0.85 (0.19-5.37) | 1.12 (0.11-3.01) | 1.71 (0.13-4.86) |
| | Swaymeh | | |
| | Late | Early | Middle |
| SP12 | 3.68 (0.07-12.28) | 1.28 (0.05-3.52) | 1.23 (0.01-9.41) |
| SP14 | 2.89 (0.35-25.54) | 0.83 (0.004-6.55) | 0.74 (0.009-7.40) |
| SP28 | 1.45 (0.03-17.86) | 0.32 (0.03-20.30) | 0.24 (0.01-5.07) |
| SP29 | 5.45 (1.79-9.99) | 1.40 (0.04-6.48) | 2.22 (0.01-9.07) |
| SP30 | 2.65 (0.32-10.53) | 0.89 (0.04-4.90) | 1.94 (0.04-7.49) |
| SP32 | 10.25 (0.003-28.57) | 3.58 (0.002-16.81) | 14.82 (0.31-5.03) |
| SP36 | 5.02 (0.97-9.11) | 0.91 (0.02-3.04) | 3.03 (0.05-11.99) |
| SP42 | 4.59 (1.29-11.94) | 0.96 (0.007-7.47) | 1.00 (0.004-5.99) |
| SP44 | 3.48 (0.33-14.96) | 0.63 (0.03-7.93) | 0.55 (0.008-4.05) |
| | North Sinai | | |
| | Late | Early | Middle |
| SP12 | 1.23 (0.01-9.41) | 2.24 (0.94-7.37) | 2.11 (0.09-6.78) |
| SP14 | 0.74 (0.009-7.40) | 1.44 (0.41-4.72) | 3.40 (0.04-17.56) |
| SP28 | 0.24 (0.01-5.07) | 2.39 (0.16-10.67) | 1.59 (0.001-6.77) |
| SP29 | 2.22 (0.01-9.07) | 2.43 (0.71-4.74) | 2.21 (0.09-11.79) |
| SP30 | 1.94 (0.04-7.49) | 1.31 (0.47-5.31) | 1.32 (0.04-3.91) |
| SP32 | 14.82 (0.31-5.03) | 1.55 (0.70-7.78) | 1.75 (0.01-7.77) |
| SP36 | 3.03 (0.05-11.99) | 1.11 (0.33-3.51) | 0.42 (0.02-3.44) |
| SP42 | 1.00 (0.004-5.99) | 1.57 (0.66-6.66) | 0.64 (0.007-3.84) |
| SP44 | 0.55 (0.008-4.05) | 1.49 (0.08-4.51) | 0.96 (0.05-6.14) |

Median gene expression is presented. Ranges are displayed in parentheses.
